# Participants stories about long-term achievement 60-months after attending a Healthy Life Centre programme (the VEND-RISK study) - a qualitative study

**DOI:** 10.1080/17482631.2022.2162984

**Published:** 2022-12-28

**Authors:** Ingrid S Følling, Karen Joramo, Anne Helvik

**Affiliations:** a Centre for Obesity Research, Clinic of Surgery, St. Olavs University Hospital, Trondheim, Norway; b Department of Clinical and Molecular Medicine, Faculty of Medicine and Health Sciences, Norwegian University of Science and Technology, Trondheim, Norway; c Department of Public Health and Nursing, Faculty of Medicine and Health Sciences, Norwegian University of Science and Technology, Trondheim, Norway

**Keywords:** Health habits, primary healthcare, dietary changes, physical activities, qualitative research

## Abstract

**Background:**

Experiences of long-term achievement in participants attending programs for changing health habits including diet and physical activity interventions aiming for weight reduction is lacking. This study aimed to explore how participants experienced their own achievement of healthy habits 60 months after attending a Healthy Life Centre programme in the Norwegian primary healthcare.

**Methods:**

An explorative qualitative design with an inductive approach was employed. Individual semi-structured interviews were performed with 20 participants attending a Healthy Life Centre programme 60 months ago. They were aged 30–72 years, and 55% were females. Interviews were transcribed verbatim and analysed thematically using systematic text condensation.

**Results:**

Two main themes emerged from the interviews with three subthemes each. The first theme “Changes over time” includes “Nutritional changes”, “Physical activities adjusted to own presumptions” and “Health habits incorporated into life with manageable goals”. The second theme “Barriers to fulfil changes” includes ”Life circumstances with health issues influences the continuity to adjust to changes”, “a busy everyday life” and “a lack of external drive when not having commitment to the Healthy Life Centre”.

**Conclusions:**

Focusing on changes adjusted to participants' own everyday lives and having an approach with small goals can facilitate long-term changes in health habits.

## Introduction

Over the last few decades, there has been an increasing global interest in enabling people to make lifestyle changes to prevent non-communicable chronic diseases (Budreviciute et al., 2020). To meet this demand in Norway, the Norwegian Directorate of Health recommends that all municipalities have Healthy Life Centres (HLCs) established in the primary healthcare to offer support to people with chronic conditions or at high risk of diseases who need to change health habits to improve health and prevent non-communicable diseases (The Norwegian Directorate of Health, 2016).

The HLCs theoretical foundation is a salutogenic approach that aims to help participants gain confidence in their own resistance resources and increase their ability to change their health habits (Langeland, 2010; The Norwegian Directorate of Health, 2016). Salutogenesis explores factors that might contribute to pushing people upwards to a good health (Antonovsky, 1987). According to the salutogenic approach, the ability to use resources is termed a Sense of Coherence (SOC). It is an individual’s way of thinking, being, and acting involving an inner trust, one which leads people to identify, benefit, use, and re-use the resources at disposal. It is demanding to perform changes in health habits, and it is a prerequisite that there is a meaning with the change, with reachable goals and having accessible resources of that one knows how to use one's resources (Antonovsky, 2010). Furthermore, an individual’s adherence to change is a continuous process and contains several different phases (Armitage, 2009). Individual barriers may be countless, and they can be hard to address by health personnel (Summerskill & Pope, 2002). Previous studies have found participants in behaviour change interventions to encounter multiple individual barriers during the process; social, psychological and practical barriers can make changing health habits hard to perform (Følling et al., 2015; Murray et al., 2012). Even if the goals for positive health outcomes are moderate and reachable, it requires substantial effort and time investment in changing health behaviours.

In order to the achieve positive health habits outcomes for participants, the duration, content and resources of HLC programmes are drawn into question (Følling, 2017). A study from HLC found that the initial improvement in physical activity levels immediately after the intervention was not maintained during the 12-month follow-up (Blom et al., 2020). Another study, the VEND-RISK study, specifically addressing type 2 diabetes risk for participants using the HLC programme as an intervention, found improved diabetes risk measures and anthropometrics at 24-months follow-up (Følling, 2017). At the 60-months follow-up of the VEND-RISK study, the 24-months follow-up results were maintained, and the type 2 diabetes and anthropometrics were further improved (Følling et al., 2022). However, there is an uncertainty in how interventions like HLC programmes actually contribute to a change in health habits and contribute to positive health outcomes (Denison et al., 2014). A qualitative study from HLCs found differences in participants' presumptions for change (Gjertsen et al., 2021). One qualitative study found participants having barriers for change (Følling et al., 2015), whilst another found participants having resources available to perform a change (Følling et al., 2016). It has been questioned who benefit from HLC programmes (Følling et al., 2015), and especially in the long term. The knowledge on participants’ own experiences of their long-term achievement after attending HLC programmes is lacking. Hence, this study aimed to explore participants’ experiences of achieving changing health habits 60 months after attending the VEND-RISK Study.

## Methods

### Design

This study employed an explorative qualitative design with an inductive approach (Doyle et al., 2020).

### Recruitment and participants

Those included in the VEND-RISK study were individuals aged >18 years with a high risk for developing type 2 diabetes (FINDRISC score > 12) and a BMI > 25 kg/m^2^ living in four municipalities (Stjørdal, Meråker, Tydal and Selbu) in Central Norway. They were recruited through an advertisement in a local newspaper and by referrals from general practitioners (GPs). Altogether, four groups were included at four time points from 2010 to 2013, thus, the first group completed the 60-months follow-up of the VEND-RISK study in 2015 and the last in 2018.

The present qualitative study included informants in 2017 (n = 27). Eligible participants for inclusion should have completed the 60-months-follow-up in the VEND-RISK study. They all got an information letter about the study by mail one week after a research coordinator at the Centre for Obesity Research, St. Olav’s Hospital, phoned them asking if they wanted to be interviewed. A variation in informants age, education level and work status was desired. First, 14 informants were recruited, but because few males were among them. Thereafter, there was a desire to recruit more males. Recruitment continued until saturation was met. Altogether, 20 participants were interviewed, all Caucasian, aged between 30 and 72 years, with 55% being females (Table 1).

**Table I.** Informant characteristics.

Characteristics	Total (N = 20)
Sociodemographic variables	N
Gender	
*Females*	11
*Males*	9
Age	
*30-39*	3
*40-49*	4
*50-59*	4
*60-69*	5
*70*	4
Civil status	
*Partner/Married*	16
*Single/Separated*	4
Highest level of education	
*Nine years of school*	13
*Bachelor’s degree or higher*	7
Work status	
*Working 100%*	12
*On disability leave*	3
*Retired*	5
Weight-related comorbidities	
*Type 2 diabetes*	4
*Cardiovascular diseases*	9
*Muscle-skeletal diseases*	3
*Psychiatric diseases*	3
*Other diseases*	5

### Interviews

Interviews were conducted between September and December 2017 at suitable locals in the Municipalities of Stjørdal, Selbu and Meråker. The nurse working at the HLC in Værnesregionen helped in facilitation of the interviews.

The first author (ISF) performed the first three interviews, while the second author (KJ) performed the rest of the interviews. An audio recorder was used, and field notes were made during each interview. The interviews had a mean duration time of 46 min (24–92 min). A semi-structured interview guide was employed, and the main questions with probes are cited in Table 2.

**Table II.** The main questions and probes from the semi-structured interview guide.

Main Questions	Probes
*«What was your main reason for attending the VEND-RISK Study?”*	*Was it yourself that initiated to participate?* *What did you want to achieve?* *What was it due to that you wanted a change?*
*“Could you tell me a little about attending the VEND-RISK Study and what it has meant to you?”*	*For your change and achievement?* *For your goals?*
*“Could you say something about the changes you did or not did?”*	*Nutritional* *Physical activity* *How did it fit with your daily life?*
*“How has it been after you finished the study?”*	*With your family, work and social?* *For the goals you had set?* *Do you still have goals to achieve?*
*“What do you experience as important for the achievement of changing health habits?”*	*And for achieving a long and lasting change?*

Follow-up questions and probes were used to clarify and explore what they considered to be important.

### The VEND-RISK study

The VEND-RISK study was initiated in 2010 by the Centre for Obesity Research at St. Olavs Hospital in cooperation with four municipalities in central Norway, aiming to prevent type 2 diabetes, overweight and obesity.

VEND-RISK purposely intended using existing local interventions in the primary healthcare to evaluate “real-life” interventions for the outcome on type 2 diabetes risk. Hence, the study offered participants the opportunity to attend the established HLC programme in their municipality. Additionally, by participating in the VEND-RISK Study evaluating the type 2 diabetes risk, they were invited to annual follow-up measurements for 60 months. These measurements were anthropometrics’ with weight and waist circumference, blood samples and a VO2-max test (Følling et al., 2022).

### The HLCs programme

The HLCs are shaped at the municipality level depending on local framework conditions and priorities, local resources and executive work (The Norwegian Directorate of Health, 2011). The HLCs serve as a low-threshold health service and there is no cost of participation. The period of the HLC programme lasts for 12 months. The personnel in the HLC were two nurses, whereas one was a study nurse, two contracted physiotherapists, and a part-time clinical nutritionist.

The HLC programme included both individual counselling and group-oriented dietary and physical activity offers as well as individual health conversation (The Norwegian Directorate of Health, 2011).

The group-based nutrition course, “Good food for better health”, developed by the Norwegian Directorate of Health, was offered once every period of 12 weeks (The Norwegian Directorate of Health, 2011). The course went over four weeks with two-hour sessions (a total of 10 hours), both theoretical and practical with different themes for each session. The course's aim was to inspire healthy food choices and good dietary habits with the intention to be a good start to achieve sustainable changes in dietary habits. A cookbook produced for the course was handed out to all participants.

Various physical activities, optional indoor and outdoor activity classes were offered continuously over the 12 months, two to four times a week with a duration of one to two hours each. The classes were both individual and group-based and included cardio- and resistance training. In addition, the HLC personnel provided information about activities in the local municipality such as hiking possibilities, fitness clubs, swimming, exercise classes, and walking groups that participants could attend.

Health conversations were performed at the beginning of the HLC period and every third month during the 12 months. The personnel were trained to use Motivational Interviewing both for the health conversations and for the courses (Ivarsson, 2010). The personnel also provided information about activities in the local municipality such as hiking possibilities, fitness clubs, swimming, exercise classes, and walking groups that participants could attend after the programme.

### Ethics

The Regional Committee for Medical and Health research in central Norway (REK) approved the VEND RISK study (REK nr 2010/696) and participants signed an informed consent when they were included in 2010–2013. Furthermore, for this qualitative study, a change notification was obtained from REK and all informants received oral and written information about this study. Participants in the present qualitative study signed a new informed consent form prior to the interview.

### Data analysis

The second author transcribed all interviews verbatim and were main responsibility for the analyzes. The analysis was performed thematically in six steps (Braun & Clarke, 2006). In the first step, transcripts and field notes were read to obtain an overall impression of the material. A summary of transcripts and a list of preliminary themes that occurred were written. In the second step, all meaning units were extracted and sorted into codes. In the search for themes, all codes relevant to the aim were discussed by all authors in the third step. The first and last authors read three interviews each and the summary prior to the discussion. In the fourth step, the themes were reviewed, checking that each theme worked in relation to the codes and related data. Some themes were discarded, and some codes were revised returning to steps two and three. In the fifth step, all authors defined and named the themes after exploring: *“what did this theme tell us?”* and *“how did each story fit the overall story of the data?”* Naming the themes finalized this step. In the sixth and final step, the results were written, including quotes that highlight informants’ stories. The authors met repeatedly between October 2017 and March 2018, discussing themes and possible underlying patterns in the data.

All quotes were translated from Norwegian into English.

## Results

**Figure 1.**
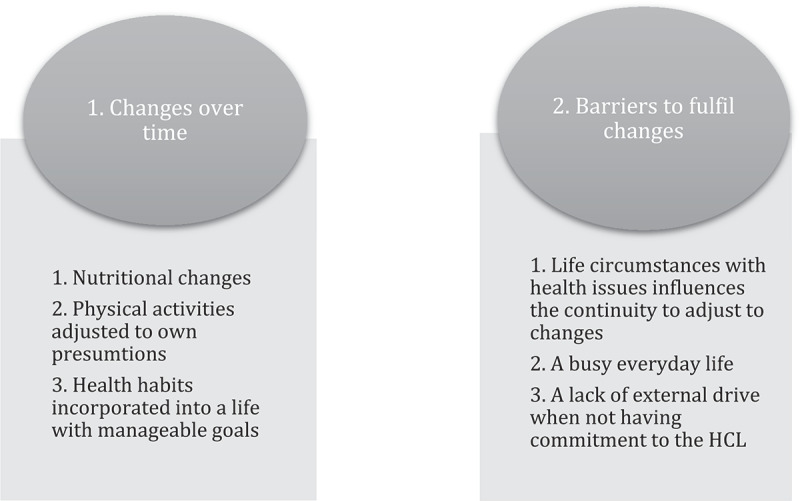
Main themes and subthemes from the informants’ stories.

Two main themes emerged from the informants’ stories (Figure 1), whereas both the first and second main themes contained three subthemes each.

### Main theme 1: changes over time

The informants said that their engagement in the program varied under their participation, and they described a different degree of achievement with reaching their goals for changes in health habits.

#### Nutritional changes

Being more conscious about what they ate and how much and the importance of small changes over time was highlighted by the participants. Informants who were parents said that they were concerned about eating healthier and having better routines for the meals, as they wanted to transfer a healthy lifestyle to their children.

*“I want my children to grow up having a healthy diet eating vegetables and fruits, and not having frozen pizza and eating candy several times a week”*. *Male 40-49 years.*

Informants talked about having performed different nutritional changes such as eating more often and reducing portion sizes and eating more vegetables. They also highlighted reducing the intake of foods high in sugar and sodium, such as sugar-beverages, chocolate, ice cream, chips and cookies. As one expressed:

*“I love baking, but I have understood that I do not need to bake three times a week and eat it all myself. I also have not been good at eating vegetables, and that is still a process for me. But mainly I have reduced eating sweets, cookies, and chips, all of that is minimized”. Female 30–39 years.*

There were different stories about how small changes in nutrition had resulted in positive changes in weight or maintenance of weight, preventing more weight gain, reducing blood sugar levels and hypertension, increased physical activity, having more daily profit and being more satisfied.

*“After one year I lost 15 kg, by doing some small changes, reducing sweetened beverages, and eating more vegetables for dinner. Somewhat I gained a few kilos, but not much, and then I further lost those kilos and now I haven’t gained more the last three years” Male 50-59 years.*

However, there was a large variation of the results of their changes from their stories. Some said that they had or almost had reached their weight loss goals, while others had not reached the weight they wanted.

#### Physical activities adjusted to own presumptions

In the participants' stories, they were concerned about being enough physical active and they had found ways to exercise that was customized to themselves. Some participants also talked about having had sport injuries, back pain and osteoarthritis, but that did not allow this to stop them from activities rather that their exercise was customized with help from a physical therapist or others at the gym.

The amount and type of exercise could vary; thus, they adjusted their exercise to their health condition, preference and time schedule. When they had started the VEND-RISK programme they had signed up for interval training and the other activities offered. They said that from prior to start up they had increased their daily living activities, like choosing the stairs instead of the elevator, walk or bicycle to the grocery store instead of driving, and they also had started walking more in general. It was especially highlighted that hiking was the main activity that was increased and which they had continued with after the programme. Hiking varied from going up the mountains to being in the forest with children and grandchildren. They also reported that several from the programme met regularly and went hiking together. As one participant said:

*“We are now many that go hiking here in the mountains nearby and we enjoy each other’s company. We consist of a spectre with people from some that are in a very bad physical shape while others are in a very good shape and nevertheless it does not matter. We stop and wait, and then it goes well. It is motivating and fun!” Female 40–49 years.*

#### Health habits incorporated into life with manageable goals

In the participants’ stories, it was repeated that it was important to make a change that was possible to incorporate in their daily life. They talked about being strived to have goals that were manageable, and they pointed out that if they were going to make several changes the risk of failing was higher. Some said they had small goals that they still made for themselves. They planned to go hiking and/or bicycling a couple of times during a week, which were manageable for them. However, it was not always easy to implement what they had planned. Others said they had set realistic goals for weight reduction and that they were more focused on their physical health and shape than the kilograms. One said that she had decreased the claims she had put in for weight reduction when she had started the programme, as she had realized that the body changes with age and hormonal changes. She underlined the importance of not having to achieve high goals by expressing:

*“You can do almost anything in a short time if you want, but you must find some changes that you can implement in your everyday life. Like in 20 years, I can still feel that I need to go hiking once a week. It is about finding something that suits you, and that will continue to do that the rest of your life”. Female 40–49 years.*

### Main theme 2: barriers to fulfil changes

#### Life circumstances with health issues influences the continuity to adjust to changes

The participants emphasized that circumstances in their life could stop them from eating healthy and exercise. Unforeseen events and their health conditions, temporary illnesses, back pain, problems related to their work, challenges during and after pregnancy, could be barriers to performing changes as planned. More pronounced among females in the study was that they talked about psychological challenges, low self-esteem and previous failure to change made it hard to have belief in a long-lasting change. Females also mentioned that candy and sweets were used as a comfort to suppress feelings of having failed to reach their goal. Some informants experienced that their body countered the changes they performed as metabolic resistance due to age and genes.

*“I have during my adult life had psychological challenges that have made it hard for me to focus on nutrition and physical activity. There are good periods when I believe in the possibility to change, but it is like my genes make it harder for me” Female 50–59 years.*

*A busy everyday life*

Another barrier that was mentioned was a busy everyday life. Some participants had hobbies and occupations in their spare time that made it hard to have time to exercise, something they wanted to prioritize differently prospectively. Nevertheless, it was hard to make this a priority. Other obstacles in their everyday life were long working days, having children that were active with their hobbies. Those with children of low age said that they did not have the possibility to exercise whenever they wanted, and they were going to exercise when the children became older. Simultaneously they also told that they went on hiking with the children and exercised at home when they experienced to have time for it, as one expressed:

*“It is easy to find excuses all the time, but it is also clear when you have children and there is a time squeeze, if you are going to exercise it will be at the expenses of something else. Thus, if there is no football matches or other activities with the children in the weekends we go skiing, fishing, hiking or are active with the kids. That is something we are holding on to every weekend”. Male 40–49 years*

#### A lack of external drive when not having commitment to the HCL

Most participants expressed how the importance of being part of the VEND-RISK study with the annual assessments contributed to an extra effort towards a healthier lifestyle. They said that it was hard to manage the changes after the VEND-RISK support ended, and they became on their own. Those who had been most active at the HLC programmes activity talked about the decrease in amount of exercise when they did not have HLC personnel that lead them after the programme was finished. They argued that they did not exercise at the same level as when some external pushed them at an exercise class, and when being in the study, they felt obliged to attend the exercise classes. However, some expressed they had quit exercising or reducing the amount or changed to go hiking instead.

It was also revealed in their stories that they pulled themselves together before they went to the annual follow-up in the VEND-RISK study containing a health conversation and several tests.

*It is not the same after the study finished, both with the exercise in the groups, but also having the annual physical tests was a motivator for me to be more active. When I knew it was time for the annual test, I worked out a little bit more the months and weeks before. Male 50-59 years.*

They said that their physical performance was displayed after the testing. Some also pointed out the importance of being weighted during health conversations, since they would not allow themselves to gain weight between the annual assessments. One said:

*“For me to come to the HLC and meet one of the health personnel and be weighted had everything to say to keep up my motivation for change.” Female 30–39 years.*

They all highlighted the aspect of being in charge and responsible for the changes themselves, but it was much easier with someone who monitored them as was done in the study. One emphasized this: *“it was a little like someone held me in the nape of the neck”. Female 60–69 years.*

## Discussion

This study explored how participants described their achievements of change towards healthier habits, and how they experienced change during and after attending the VEND-RISK Study, which included HLC intervention. The main results presented were two themes: *Changes over time* and *Barriers to fulfil changes*. When participants spoke about changes over time, it consisted of nutritional changes, physical activities adapted after their own requirements and how their health habits were incorporated into life with manageable goals. Talking about the barriers to the adapted changes, they highlighted that life circumstances with health issues influence the continuity to the adapted changes, a busy everyday life and that they experienced a lack of external drive after ending participation in VEND-RISK.

In our results, the participants expressed that it was hard to comply with the recommendations for physical activity with moderately 150 min/week or 75 min/week with high intensity, but they emphasized that some exercise was better than none. They adapted the exercise after their everyday-life situation. Going for walks was something they highlighted as something they continued with after they ended their participation in VEND-RISK. In addition, our study results described the participants’ social network of importance for continuing with walks. Previous results have shown that going from being inactive to more active, i.e., to half of the level of the recommendations of 150 min/week, could reduce the risk for non-communicable diseases and mortality significantly (Warburton & Bredin, 2016). This is in line with results from the VEND-RISK study, where participants had improved diabetes risk measures and anthropometrics at 24-months follow-up (Følling, 2017) and continued to have a decreased risk at 60 months follow-up (Følling et al., 2022). However, for weight-loss maintenance, there is recommended to exercise more than the moderate 150 min/week, and there is shown an association between exercising 60–90 min a day with a better maintenance of weight loss (Swift et al., 2014). Thus, there is reported a need for more experimental studies with long time follow-up to investigate maintenance of weight-loss (Alamuddin & Wadden, 2016). The largest Norwegian study of HLC participants to date found that the initial improvement in physical activity levels immediately after the intervention was not maintained at 12 months follow-up (Blom et al., 2020). Results from another HLC study found that over half of the respondents had increased their activity level two to four years after ending participation (Bratland-Sanda et al., 2014). Regardless of the inconclusiveness from previous results, having exercise in an intervention could be advantageous in regard to social support and the commitment (Artinian et al., 2010; Bratland-Sanda et al., 2014), as also participants in our study reported. Furthermore, the element of physical activity as part of a healthy lifestyle could help individuals to become more secure and result in an overall better well-being (Richards et al., 2015).

Participants in the present study emphasized the importance of implementing healthy habits that were suited to their everyday life. There were a difference between the participants in degree of achievement of their goals for healthy habits. They had set reachable goals for exercise and nutritional changes that they meant were possible to maintain over time, focusing on small changes for long-lasting changes. Another study reported that participants not managing dietary changes in line with recommended guidelines justified this with unrealistic requirements and changes not fitting their life (Thomas et al., 2008). Having realistic goals fitting their abilities is associated with better success with behavioural and lifestyle interventions (Artinian et al., 2010; Klein et al., 2004). Nevertheless, it is of importance to set goals that are high enough. When implementing goals, they may contribute to a feeling of satisfaction when they are achieved (Artinian et al., 2010). A presumption for change is to experience a meaning with performing the change, and consequently, would find it is worth engaging in doing necessary activities to reach their goals. Having adequate help and guidance from health personnel when starting behaviour change is also important (Rise et al., 2013). Health personnel can contribute to a more comprehensible situation for the participant, drawing attention to resources and assist and support the participants to customize their goals to be in line with their capacity (Walseth & Malterud, 2004). Nevertheless, in general, persons with a strong SOC have a higher possibility of making choices that may promote their health (Antonovsky, 2010). Studies have found strong associations’ between SOC and better physical health and healthy lifestyle (Eriksson & Lindström, 2006; Wainwright et al., 2007) and that persons at risk for type 2 diabetes with a stronger SOC had better outcomes for lifestyle interventions than those with lower SOC (Nilsen et al., 2015). Reasoned in the findings and the theory, it is likely that a closer follow-up over several years could be more helpful for some HLC attenders, as their capacity differs; their needs for follow-up are also different. For some HLC attendees, it could be enough to be weighted frequently, whilst others need a closer and more detailed plan for implementing changes and have health personnel empowering them to perform such a plan. For those who are in need of a closer follow-up, actions/interventions to strengthen their SOC, it could contribute to more positive health outcomes (Nilsen et al., 2015) during and after the HLC programme. A possible solution to find out more about the participants' individual needs could be to measure their SOC.

### Strengths and limitations

Regarding the qualitative design of this study, we cannot generalize our findings to all participants in HLCs. Even so, there is a strength in having several informants with a variation in age, education level and work status. We believe our results to be potentially transferable to participants aiming for health behaviour change. Thus, our study adds new knowledge on this field.

The participants interviewed in this study had all completed the 60 months follow-up in the VEND-RISK study, which meant that they could be more positive than those not having completed. They also agreed to be interviewed that also may indicate that they were positive about the study and their attendance.

Another consideration for our study is that the participants change in health partly might have occurred due to the study participation and the follow-up including to be seen by the personnel and cared for in the study, i.e., the “Hawthorne effect” (Ho et al., 2008). Though if this effect could have been present, our study explored their experiences and the results showed that they lacked an external stimulus when the study ended that could be what they were being observed in the study setting. This may indicate that there is a positive health outcome to being observed and given attention and follow-up stimuli and that for people in behavioural intervention being followed up is of importance.

## Conclusion

The present results found two main themes. The first theme was *Changes over time* including nutritional changes, physical activities adapted after own requirements and how health habits were incorporated into life with manageable goals. The second theme was *Barriers to fulfil change*, including life circumstances with health issues influencing the continuity to the adapted changes, a busy everyday life and the fact that they experienced a lack of external drive after ending participation in the study. These results show the importance of making participants in HLCs programmes more aware of adjusting the changes based on the individuals’ life situation. Furthermore, a focus on small changes could have positive health effects that seem more manageable and could contribute to a desire to maintain the changes and continue further change.

## Data Availability

The raw data supporting the findings in this article can be found at the Centre for Obesity Research (ObeCe), St. Olavs Hospital, Trondheim, Norway. Due to the Regional Committee for Medical and Health Research Ethics in Central Norway regulations, we have to secure the anonymity of the participants. In the raw data, it is possible to identify the participants, and restrictions therefore apply to the availability of these data.

## References

[cit0001] Alamuddin, N., & Wadden, T. A. (2016). Behavioral Treatment of the Patient with Obesity. *Endocrinology and Metabolism Clinics of North America*, 45(3), 565–9. 10.1016/j.ecl.2016.04.008 27519131

[cit0002] Antonovsky, A. (1987). In *Unraveling the mystery of health: how people manage stress and stay well* (Jossy-Bass) 218 .

[cit0003] Antonovsky, A. (2010). *Sjøbu A. Helsens mysterium: undefined salutogene modellen. 2012*. Gyldendal akademisk.

[cit0004] Armitage, C. J. (2009). Is there utility in the transtheoretical model? *British Journal of Health Psychology*, 14(Pt 2), 195–210. 10.1348/135910708X368991 18922209

[cit0005] Artinian, N. T., Fletcher, G. F., Mozaffarian, D., Kris-Etherton, P., Van Horn, L., Lichtenstein, A. H., Kumanyika, S., Kraus, W. E., Fleg, J. L., Redeker, N. S., Meininger, J. C., Banks, J., Stuart-Shor, E. M., Fletcher, B. J., Miller, T. D., Hughes, S., Braun, L. T., Kopin, L. A., Berra, K. … American Heart Association Prevention Committee of the Council on Cardiovascular Nursing. (2010). Interventions to promote physical activity and dietary lifestyle changes for cardiovascular risk factor reduction in adults: A scientific statement from the American Heart Association. *Circulation*, 122(4), 406–441. 10.1161/CIR.0b013e3181e8edf1 20625115 PMC6893884

[cit0006] Blom, E. E., Aadland, E., Skrove, G. K., Solbraa, A. K., & Oldervoll, L. M. (2020). Health-related quality of life and physical activity level after a behavior change program at Norwegian healthy life centers: A 15-month follow-up. *Quality of Life Research : An International Journal of Quality of Life Aspects of Treatment, Care and Rehabilitation*, 29(11), 3031–3041. 10.1007/s11136-020-02554-x 32562195 PMC7591434

[cit0007] Bratland-Sanda, S., Lislevatn, F., & Lerdal, A. (2014). Healthy Life Centre Recept- a prevalence study from Modum Municipality Healthy Life Centre. *Fysioterapeuten*, 3 (14), 18–24.

[cit0008] Braun, V., & Clarke, V. (2006). Using thematic analysis in psychology. *Qualitative Research in Psychology*, 3(2), 77–101. 10.1191/1478088706qp063oa

[cit0009] Budreviciute, A., Damiati, S., Sabir, D. K., Onder, K., Schuller-Goetzburg, P., Plakys, G., Katileviciute, A., Khoja, S., & Kodzius, R. (2020). Management and Prevention Strategies for Non-communicable Diseases (NCDs) and Their Risk Factors. *Frontiers in Public Health*, 8, 574111. 10.3389/fpubh.2020.574111 33324597 PMC7726193

[cit0010] Denison, E., Vist, G. E., Underland, V., & Berg, R. C. (2014). In *Effects of More than Three Months Organized Follow-Up on Physical Activity and Diet for People with Increased Risk of Lifestyle Disease*. Knowledge Centre for the Health Services at The Norwegian Institute of Public Health (NIPH). 29320070

[cit0011] Doyle, L., McCabe, C., Keogh, B., Brady, A., & McCann, M. (2020). An overview of the qualitative descriptive design within nursing research. *Journal of Research in Nursing : JRN*, 25(5), 443–455. 10.1177/1744987119880234 34394658 PMC7932381

[cit0012] Eriksson, M., & Lindström, B. (2006). Antonovsky’s sense of coherence scale and the relation with health: A systematic review. *Journal of Epidemiology and Community Health*, 60(5), 376–381. 10.1136/jech.2005.041616 16614325 PMC2563977

[cit0013] Følling, I. S. (2017). Participants in Healthy Life Centre’s presumptions for lifestyle change: Preventing overweight, obesity and type 2 diabetes in the Norwegian Primary Health Care (NTNU).

[cit0014] Følling, I. S., Klöckner, C., Devle, M. T., & Kulseng, B. (2022). Preventing type 2 diabetes, overweight and obesity in the Norwegian primary healthcare: A longitudinal design with 60 months follow-up results and a cross-sectional design with comparison of dropouts versus completers. *BMJ Open*, 12(3), e054841. 10.1136/bmjopen-2021-054841 PMC8915299 35264353

[cit0015] Følling, I. S., Kulseng, B., Midthjell, K., Rangul, V., & Helvik, A. S. (2017). Individuals at high risk for type 2 diabetes invited to a lifestyle program: Characteristics of participants versus non-participants (the HUNT Study) and 24-month follow-up of participants (the VEND-RISK Study). *BMJ Open Diabetes Research & Care*, 5(1), e000368. 10.1136/bmjdrc-2016-000368 PMC5574427 28878932

[cit0016] Følling, I. S., Solbjør, M., & Helvik, A. S. (2015). Previous experiences and emotional baggage as barriers to lifestyle change - a qualitative study of Norwegian Healthy Life Centre participants. *BMC Family Practice*, 16(1), 73. 10.1186/s12875-015-0292-z 26100276 PMC4476174

[cit0017] Følling, I. S., Solbjør, M., Midthjell, K., Kulseng, B., & Helvik, A. S. (2016). Exploring lifestyle and risk in preventing type 2 diabetes-a nested qualitative study of older participants in a lifestyle intervention program (VEND-RISK). *BMC Public Health*, 16(1), 876. 10.1186/s12889-016-3559-y 27557801 PMC4997726

[cit0018] Gjertsen, T. I., Helvik, A. S., & Følling, I. S. (2021). Previous life experiences and social relations affecting individuals wish for support when establishing healthy habits - a qualitative study of Norwegian Healthy Life Centre participants. *BMC Public Health*, 21(1), 1315. 10.1186/s12889-021-11374-8 34225666 PMC8256571

[cit0019] Ho, P. M., Peterson, P. N., & Masoudi, F. A. (2008). Evaluating the evidence: Is there a rigid hierarchy? *Circulation*, 118(16), 1675–1684. 10.1161/CIRCULATIONAHA.107.721357 18852378

[cit0020] Ivarsson, B. H. (2010). *MI Motiverende Samtaler* (Gyldendal akademisk) 120.

[cit0021] Klein, S., Sheard, N. F., Pi-Sunyer, X., Daly, A., Wylie-Rosett, J., Kulkarni, K., Clark, N. G., & American Diabetes Association, North American Association for the Study of Obesity, & American Society for Clinical Nutrition. (2004). Weight management through lifestyle modification for the prevention and management of type 2 diabetes: Rationale and strategies. A statement of the American Diabetes Association, the North American Association for the Study of Obesity, and the American Society for Clinical Nutrition. *The American Journal of Clinical Nutrition*, 80(2), 257–263. 10.1093/ajcn/80.2.257 15277143

[cit0022] Langeland, E. (2010). The meaning of a salutogenic approach to promote psychic health. *Nursing Research*, 04, 289–296.

[cit0023] Murray, J., Craigs, C. L., Hill, K. M., Honey, S., & House, A. (2012). A systematic review of patient reported factors associated with uptake and completion of cardiovascular lifestyle behaviour change. *BMC Cardiovascular Disorders*, 12(1), 120. 10.1186/1471-2261-12-120 23216627 PMC3522009

[cit0024] Nilsen, V., Bakke, P. S., Rohde, G., & Gallefoss, F. (2015). Is sense of coherence a predictor of lifestyle changes in subjects at risk for type 2 diabetes? *Public Health*, 129(2), 155–161. 10.1016/j.puhe.2014.12.014 25682903

[cit0025] The Norwegian Directorate of Health. (2011). National Instruction Manual for municipal Healthy Life Centres. In *The Norwegian Directorate of Health*. Oslo.

[cit0026] The Norwegian Directorate of Health. (2016). In *Guidelines for Municipal Healthy Life Centers*.

[cit0027] Richards, J., Jiang, X., Kelly, P., Chau, J., Bauman, A., & Ding, D. (2015). Don’t worry, be happy: Cross-sectional associations between physical activity and happiness in 15 European countries. *BMC Public Health*, 15(1), 53. 10.1186/s12889-015-1391-4 25636787 PMC4320474

[cit0028] Rise, M. B., Pellerud, A., Rygg, L. Ø., Steinsbekk, A., & Jenkins, N. (2013). Making and maintaining lifestyle changes after participating in group based type 2 diabetes self-management educations: A qualitative study. *PLoS Onene*, 8(5), e64009. 10.1371/journal.pone.0064009 PMC3650057 23671705

[cit0029] Summerskill, W. S., & Pope, C. (2002). ‘I saw the panic rise in her eyes, and evidence-based medicine went out of the door.’ an exploratory qualitative study of the barriers to secondary prevention in the management of coronary heart disease. *Family Practice*, 19(6), 605–610. 10.1093/fampra/19.6.605 12429662

[cit0030] Swift, D. L., Johannsen, N. M., Lavie, C. J., Earnest, C. P., & Church, T. S. (2014). The role of exercise and physical activity in weight loss and maintenance. *Progress in Cardiovascular Diseases*, 56(4), 441–447. 10.1016/j.pcad.2013.09.012 24438736 PMC3925973

[cit0031] Thomas, S. L., Hyde, J., Karunaratne, A., Kausman, R., & Komesaroff, P. A. (2008). “They all work…when you stick to them”: A qualitative investigation of dieting, weight loss, and physical exercise, in obese individuals. *Nutrition Journal*, 7(1), 34. 10.1186/1475-2891-7-34 19025661 PMC2607302

[cit0032] Wainwright, N. W., Surtees, P. G., Welch, A. A., Luben, R. N., Khaw, K. T., & Bingham, S. A. (2007). Healthy lifestyle choices: Could sense of coherence aid health promotion? *Journal of Epidemiology and Community Health*, 61(10), 871–876. 10.1136/jech.2006.056275 17873222 PMC2652963

[cit0033] Walseth, L. T., & Malterud, K. (2004). Salutogenese og empowerment i allmennmedisinsk perspektiv [Salutogenesis and empowerment in the perspective of general practice]. *Tidsskrift for den Norske Laegeforening : Tidsskrift for Praktisk Medicin, Ny Raekke*, 124(1), 65–66. 14716399

[cit0034] Warburton, D. E., & Bredin, S. S. (2016). Reflections on Physical Activity and Health: What Should We Recommend? *The Canadian Journal of Cardiology*, 32(4), 495–504. 10.1016/j.cjca.2016.01.024 26995692

